# Molecular characterisation of *Entamoeba histolytica* UDP-glucose 4-epimerase, an enzyme able to provide building blocks for cyst wall formation

**DOI:** 10.1371/journal.pntd.0011574

**Published:** 2023-08-24

**Authors:** Anna Nagode, Jorick Vanbeselaere, Zuzanna Dutkiewicz, Samantha Kaltenbrunner, Iain B. H. Wilson, Michael Duchêne

**Affiliations:** 1 Institute of Specific Prophylaxis and Tropical Medicine, Center for Pathophysiology, Infectiology and Immunology, Medical University of Vienna, Vienna, Austria; 2 Department of Chemistry, Universität für Bodenkultur, Vienna, Austria; University of Texas at El Paso, UNITED STATES

## Abstract

In the human host, the protozoan parasite *Entamoeba histolytica* is adapted to a non-invasive lifestyle in the colon as well as to an invasive lifestyle in the mesenterial blood vessels and the liver. This means to cope with bacteria and human cells as well as various metabolic challenges. Galactose and *N*-acetylgalactosamine (GalNAc) are sugars of great importance for the amoebae, they attach to the host mucus and enterocytes via their well-studied Gal/GalNAc specific lectin, they carry galactose residues in their surface glycans, and they cleave GalNAc from host mucins. The enzyme UDP-glucose 4-epimerase (GalE) works as a bridge between the galactose and glucose worlds, it can help to generate glucose for glycolysis from phagocytosis products containing galactose as well as providing UDP-galactose necessary for the biosynthesis of galactose-containing surface components. *E*. *histolytica* contains a single *galE* gene. We recombinantly expressed the enzyme in *Escherichia coli* and used a spectrophotometric assay to determine its temperature and pH dependency (37°C, pH 8.5), its kinetics for UDP-glucose (K_m_ = 31.82 μM, V_max_ = 4.31 U/mg) and substrate spectrum. As observed via RP-HPLC, the enzyme acts on UDP-Glc/Gal as well as UDP-GlcNAc/GalNAc. Previously, *Trypanosoma brucei* GalE and the bloodstream form of the parasite were shown to be susceptible to the three compounds ebselen, a selenoorganic drug with antioxidant properties, diethylstilbestrol, a mimic of oestrogen with anti-inflammatory properties, and ethacrynic acid, a loop diuretic used to treat oedema. In this study, the three compounds had cytotoxic activity against *E*. *histolytica*, but only ebselen inhibited the recombinant GalE with an IC50 of 1.79 μM (UDP-Gal) and 1.2 μM (UDP-GalNAc), suggesting that the two other compounds are active against other targets in the parasite. The importance of the ability of GalE to interconvert UDP-GalNAc and UDP-GlcNAc may be that the trophozoites can generate precursors for their own cyst wall from the sugar subunits cleaved from host mucins. This finding advances our understanding of the biochemical interactions of *E*. *histolytica* in its colonic environment.

## Author summary

*Entamoeba histolytica* is a unicellular parasite causing amoebic dysentery and liver abscess. The trophozoites live in the human colon where they feed on bacteria and simple sugars, e.g. glucose, which is essential for their fermentative energy metabolism, moreover, glucose and galactose appear in large amounts on their surface molecules. We characterised the enzyme UDP-glucose 4-epimerase (GalE). The enzyme converts UDP-galactose (UDP-Gal) to UDP-glucose (UDP-Glc), and in addition UDP-*N*-acetylgalactosamine (UDP-GalNAc) to UDP-*N*-acetylglucosamine (UDP-GlcNAc). As *E*. *histolytica* possesses a β-*N*-acetylhexosaminidase, which potentially releases GalNAc and GlcNAc from human intestinal mucins; via the action of GalE, these building blocks can be utilised to generate UDP-GlcNAc, the precursor for the chitin of the amoebic cyst wall. The compounds ebselen, diethylstilbestrol and ethacrynic acid had been reported as inhibitors of *Trypanosoma brucei* GalE and were also active against the parasite. When we tested the compounds against *E*. *histolytica* and its GalE, all three compounds led to amoebic death, but only one of them, ebselen, inhibited GalE activity. Considering its bifunctionality, GalE is not only key to the synthesis of trophozoite glycans, but probably is essential for the generation of chitin for the emerging cyst wall from sugars cleaved from the host intestinal mucin.

## Introduction

The protozoan parasite *Entamoeba histolytica* is responsible for amoebic dysentery and liver abscess in humans. A few years ago, the number of annual deaths caused by amoebiasis was estimated at 55,500 and the DALYs (years of life lost from premature death or disability) at 2.24 million [1]. This study was a partial analysis of the Global Burden of Disease 2010 study [2].

Galactose plays a role in many aspects of the pathophysiology and biochemistry of *E*. *histolytica*. The surface of the amoebic trophozoites carries the well-characterised galactose/*N*-acetylgalactosamine (Gal/GalNAc)-specific lectin. This molecule is involved in the attachment to the human intestinal mucus as well as to the enterocytes [3]. It consists of a large subunit carrying the carbohydrate-binding domain and a small glycosylphosphatidylinositol- (GPI-) anchored subunit [4]. The complex co-localises with an intermediate subunit [5]. A selected domain of the heavy chain has been proposed as a possible vaccine against amoebiasis [6].

Besides this Gal/GalNAc-specific lectin, the trophozoites also possess a surface antigen variably called lipopeptidophosphoglycan (LPPG) [7], lipophosphoglycan (LPG) [8] or proteophosphoglycan (PPG) [9], a GPI-anchored glycoprotein with a number of galactose residues in various positions [9]. This molecule is also immunogenic and a monoclonal antibody against it protected severe combined immunodeficient (SCID) mice against amoebic liver abscess [10].

The major colonic mucin, to which the trophozoites attach, is Muc2, a large O-glycosylated glycoprotein containing both *N*-acetylglucosamine (GlcNAc) and *N*-acetylgalactosamine (GalNAc) residues [11]. The Muc2 core protein chain can be cleaved by an *E*. *histolytica* cysteine proteinase, most likely by CP5 [12]. In addition, the amoebae possess two genes encoding the subunits of the heterodimeric β-*N*-acetylhexosaminidase [13], which could cleave off the terminal *N*-acetylated amino sugars from Muc2. GlcNAc could then be imported by the trophozoites to synthesize chitin when they differentiate into cysts [14].

Considering that *E*. *histolytica* N-glycans [15], not just the LPPG, contain galactose, relevant enzymes must be encoded by its genome. In the central position of a functioning galactose metabolism, the enzyme UDP-glucose 4-epimerase (GalE, EC 5.1.3.2), a member of the short chain dehydrogenase/reductase family, is needed which epimerises UDP-glucose to UDP-galactose and vice versa [16].

In mammals, GalE enzymes are necessary to generate the precursors for the sugar chains on intestinal mucins [17] and mutations in human GalE are associated with galactosaemia [18]. In the model insect *Drosophila melanogaster*, complete loss of GalE is embryonic lethal [19].

**Fig 1:**
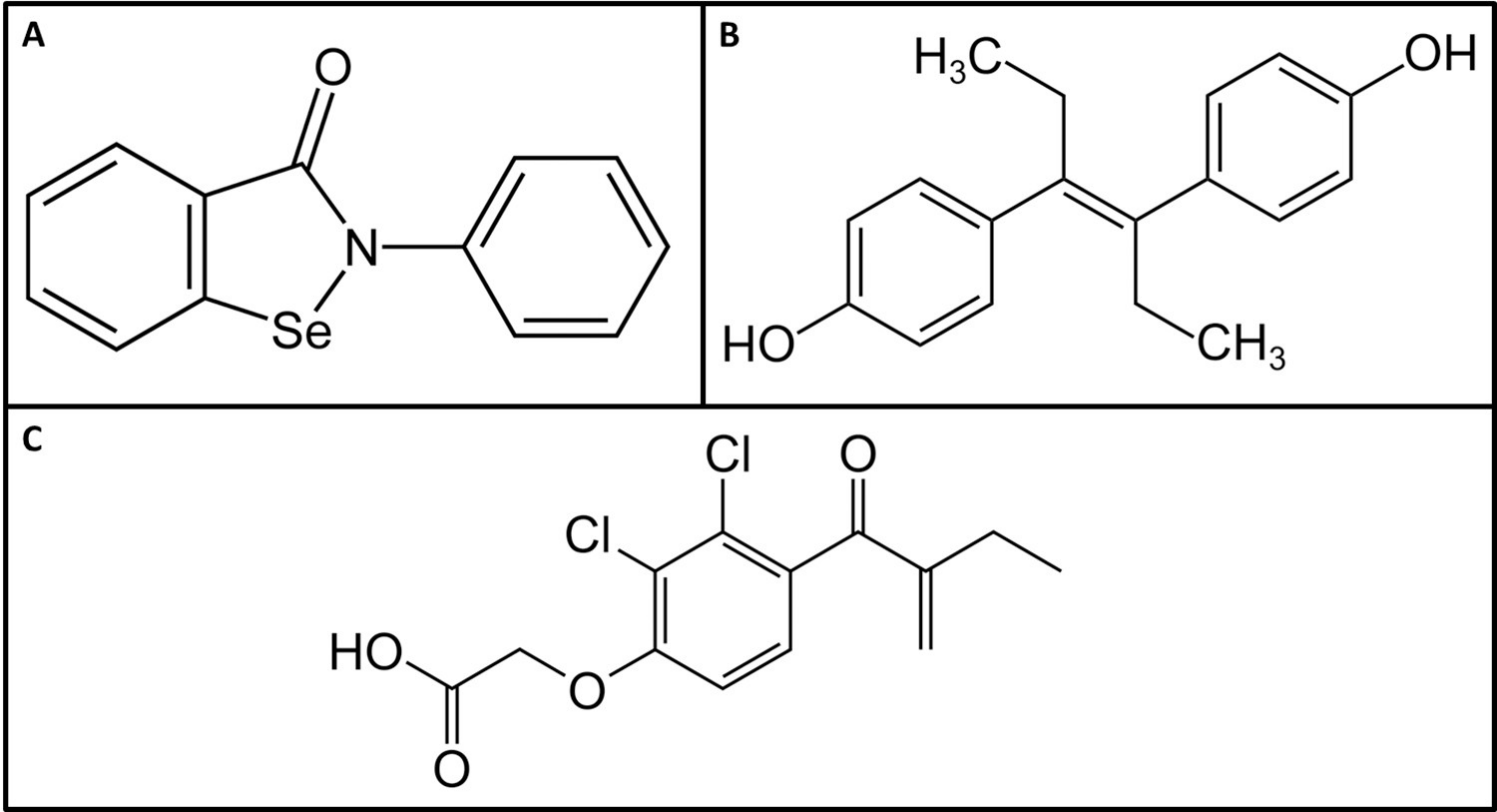
Structures of potential EhGalE inhibitors. (A) Ebselen, (B) Diethylstilbestrol (DES), (C) Ethacrynic acid (EA).

For *Trypanosoma brucei*, GalE is also essential [20], and several compounds with activity against the enzyme and the parasite were described [21]. In the present study, three of these compounds, ebselen, diethylstilbestrol (DES) and ethacrynic acid (EA) (Fig 1) were tested against *E*. *histolytica* GalE and the trophozoites. Ebselen is a well-studied selenoorganic drug with antioxidant properties, which targets cysteine thiol groups, and possesses antimicrobial and antiviral properties, notably against SARS-CoV-2 [22]. Diethylstilbestrol (DES) is a mimic of oestrogen with anti-inflammatory properties which was prescribed around the middle of last century to prevent miscarriage in pregnant women, but its use was discontinued because it increased breast cancer risk and caused other adverse effects [23]. Ethacrynic acid (EA) is a loop diuretic used to treat oedema, and inhibits glutathione S-transferase thus reducing resistance to cancer chemotherapy [24].

In this study, we cloned and characterised the single *E*. *histolytica* GalE gene and its encoded protein. The enzyme was able to epimerise UDP-Glc to UDP-Gal (EC 5.1.3.2) and we found that it could also interconvert UDP-GlcNAc and UDP-GalNAc (EC 5.1.3.7). This could enable the trophozoites to convert GalNAc cleaved from Muc2 to GlcNAc, a building block of chitin, for the transformation into a cyst, so the protective mucus layer of the colon could be rebuilt into the protective amoebic cyst wall. Furthermore, three putative GalE inhibitors were examined, of which only ebselen inhibited the GalE activity. Our results form a basis for an understanding of galactose utilisation and its incorporation into glycoconjugates in *E*. *histolytica* and propose how this parasite could utilise hexoses and *N*-acetylhexosamines released via degradation of glycans in the host colon.

## Results

### Identification and phylogeny of *E*. *histolytica* GalE

The *E*. *histolytica* genome contains a single gene coding for GalE (UDP-glucose 4-epimerase) with the accession numbers XP_650346 (NCBI Protein) or C4M746 (UniProt). To show its relatedness to other sequences, a phylogenetic analysis was performed. Homologues were identified in the Uniprot database, whereby the complication is that there is a very high number of UDP-glucose 4-epimerases annotated, but that they act on various substrates [25], and only a relatively small number are biochemically characterised.

The major substrate pair besides UDP-Glc/UDP-Gal is UDP-GlcNAc/UDP-GalNAc, and Ishiyama and colleagues grouped the enzymes in those acting on UDP-Glc/UDP-Gal only (Group 1), enzymes acting on both (Group 2), and enzymes acting on UDP-GlcNAc/UDP-GalNAc only (Group 3), with *Pseudomonas aeruginosa* WbpP assigned to group 3 [26]. We further address this classification, as applied to EhGalE, in the discussion below.

**Fig 2:**
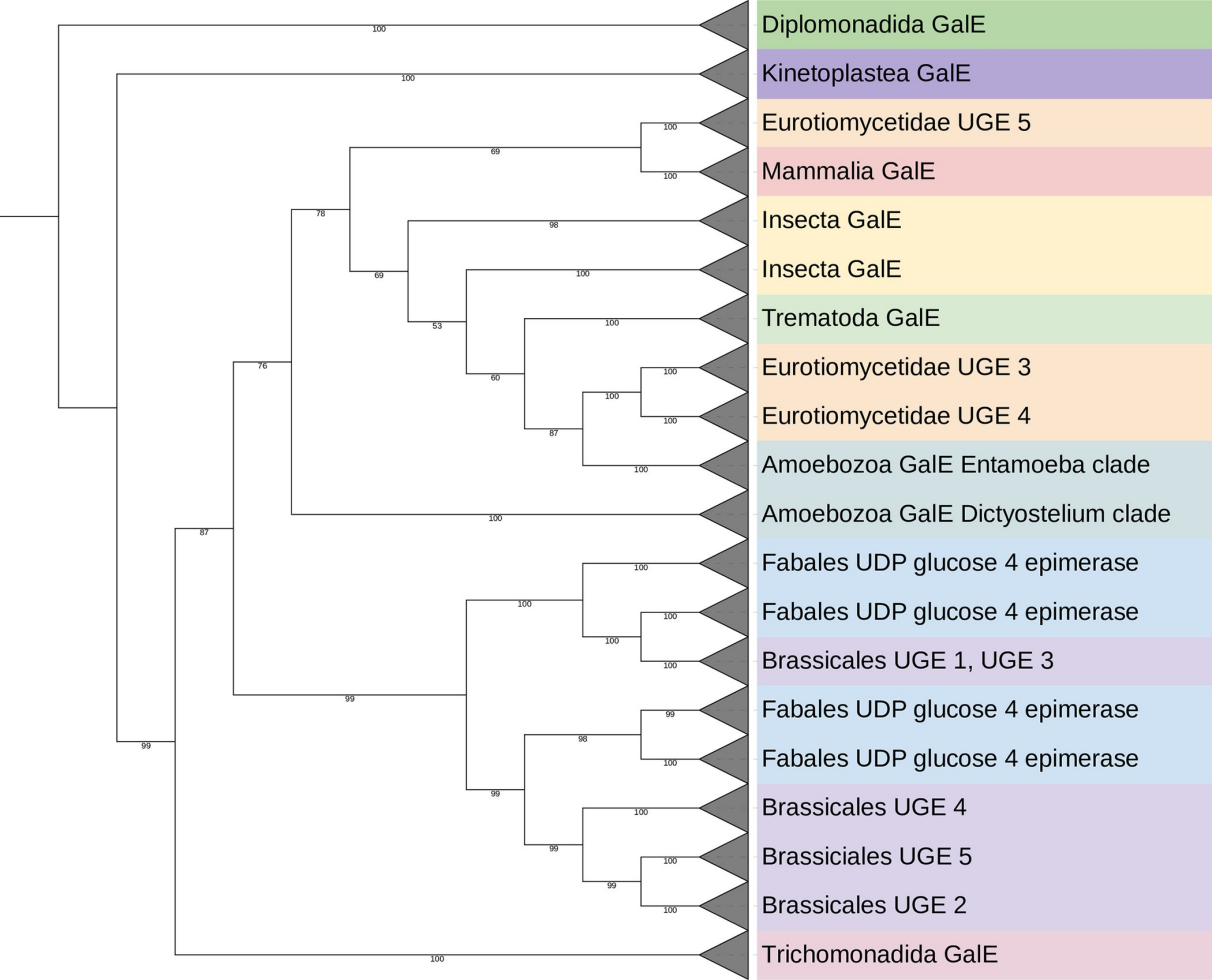
Collapsed phylogeny UDP-glucose 4-epimerase homologues. 194 protein sequences annotated as UDP-glucose 4 epimerase (abbreviated variably as GalE or UGE) were downloaded from the UniProt database [29]. The numbers below the branches indicate bootstrap support (maximum value 100). The maximum-likelihood tree was based on alignment of 764 columns with 468 parsimony-informative sites by IQTREE [30]. The LG+I+G4 model was automatically fitted by the algorithm. Alignment was done with using MAFFT [31]. The tree was rooted at midpoint and was visualized with iTOL5 [32]. The full tree is shown in S1 Fig, and an unrooted tree including bacterial sequences, aligned using MAFFT [31] and trimAl [33], is displayed in S2 Fig.

Where studied, the bacterial homologues have a complex substrate pattern. Besides the enzyme with basic activity on UDP-Glc/UDP-Gal (named GalE) or the UDP-GlcNAc/UDP-GalNAc (often designated as Gne), other activities have been described [27]. Examples are that *P*. *aeruginosa* WbpP can also accept the uronic form of the sugar [28], or WbpV annotated as UDP-Glc 4-epimerase, but which may rather be involved in UDP-QuiNAc (UDP-*N*-acetylquinovosamine) biosynthesis [28]. Considering the high diversity of GalE, Gne and WbpP sequences and partly faulty annotations, the analysis was concentrated on eukaryotic sequences (Figs 2, S1, and S2).

In the resulting tree, sequences from plants [34, 35] and fungi [36] fall into a number of different clades generally named UGE1 through to UGE5, reflecting also the different preferences of the enzymes, where these have been determined. The analysed amoebal GalE homologues fall into two different branches, the Dictyostelids and the Entamoebae, which are both closer in this tree to animal and fungal clades rather than plants or other unicellular organisms; indeed, the sequences of *Giardia* UDP-GlcNAc 4-epimerase [37] and kinetoplast UDP-Glc 4-epimerase [20] are rather distantly related.

### Expression and kinetic characterisation of *E*. *histolytica* GalE (EhGalE)

Several technologies were combined to generate recombinant EhGalE from *Escherichia coli*. The expression plasmid pET-17b with the codon usage changed from *E*. *histolytica* to *E*. *coli* (S3 Fig) was chemically synthesised. To allow easy affinity purification, we added an eight-residue sequence coding for a Strep-tag [38] at the amino-terminal end. Expression of recombinant EhGalE was performed in *E*. *coli* BL21-AI cells, followed by purification of the enzyme with a Strep-Tactin Sepharose column.

**Fig 3:**
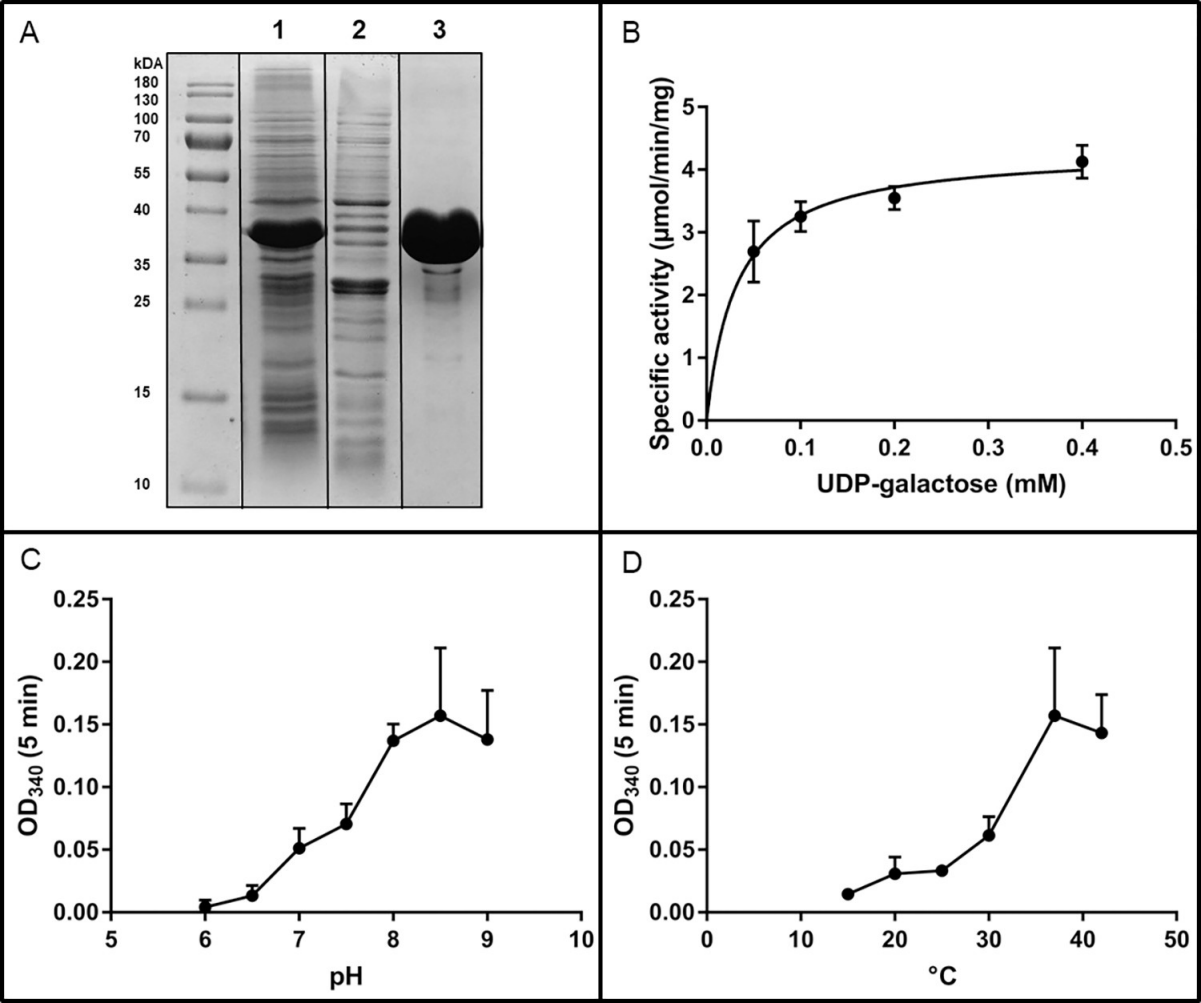
Expression and characterisation of *E*. *histolytica* GalE (EhGalE). (A) SDS-PAGE analysis of recombinant EhGalE expression and purification *Lane 1*: Crude bacterial lysate after induction of EhGalE production *Lane 2*: First wash fraction *Lane 3*: the eluted strep-tag purified EhGalE. (B) Determination of kinetic parameters of EhGalE by blotting the specific activity against increasing concentrations of the substrate UDP-galactose using the coupled assay. Calculated V_max_ of EhGalE was 4.31 ± 0.21 μmol/min/mg and K_m_ was 31.82 ± 7.65 μM at 37°C. (C) pH dependency of EhGalE was determined in MES Buffer (pH 6.0–7.0) and HEPES Buffer (pH 7.5–9.0) at 37°C. (D) Temperature dependency of EhGalE was examined at pH 8.5. All EhGalE activity measurements were performed in triplicates.

SDS-PAGE analysis showed an abundantly expressed, purified protein with a molecular mass of around 39 kDa (Fig 3A). EhGalE is predicted to be 37.8 kDa, and with the N-terminal Strep-tag and the additional residues, a molecular mass of 39.3 kDa is predicted, corresponding to the observed value.

**Fig 4:**
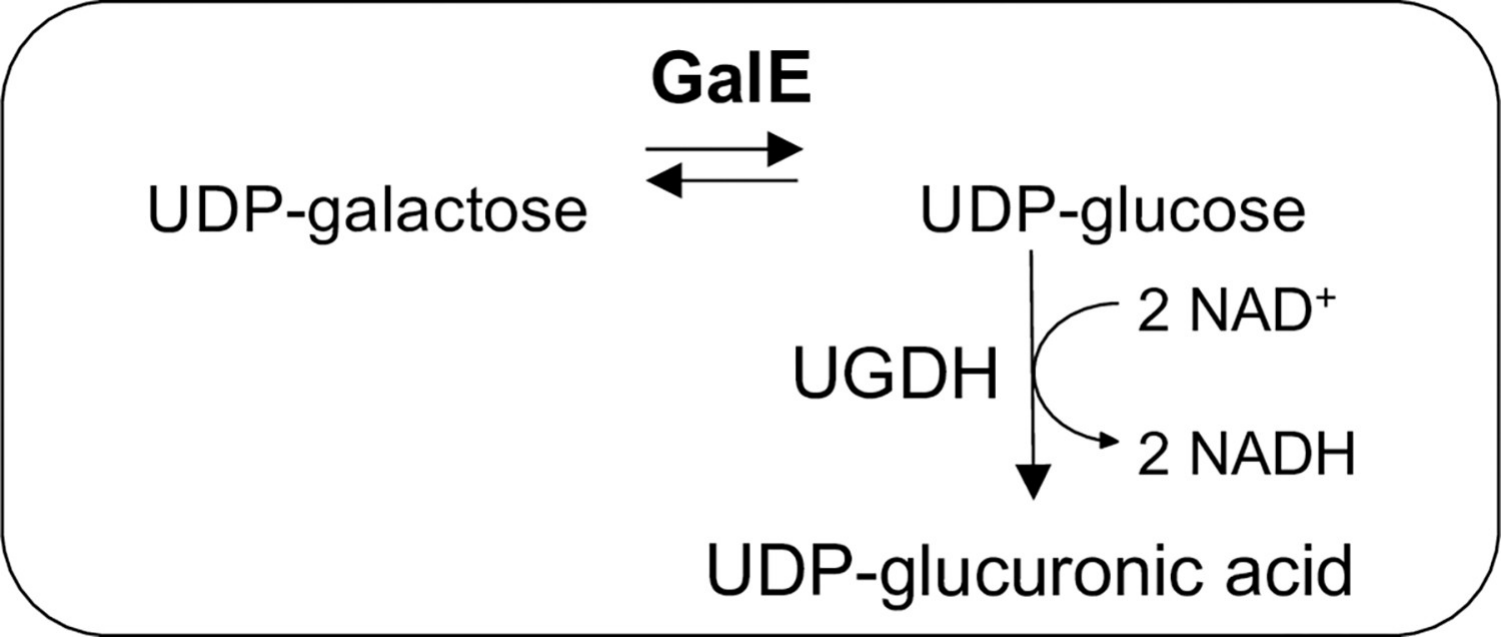
Coupled enzyme assay to determine GalE activity.

Enzymatic activity was measured indirectly, coupling the GalE reaction with UGDH (UDP-glucose dehydrogenase, EC 1.1.1.22, Fig 4). As *E*. *histolytica* does not possess a UGDH, the human enzyme was chosen and expressed and purified in the same manner as EhGalE (S4 Fig) and its activity was comparable to previous published measurements [39]. UGDH oxidises UDP-glucose to UDP-glucuronate reducing two NAD^+^ to two NADH molecules, which are observed at 340 nm in a spectrophotometer in order to determine the activity of EhGalE.

The Michaelis-Menten constant K_m_ for the substrate UDP-glucose at 37°C was determined to be 31.82 ± 7.65 μM and V_max_ was 4.31 ± 0.21 μmol/min/mg (Fig 3B). The highest EhGalE activity was observed to be at pH 8.5 (Fig 3C) and the temperature optimum of the enzyme was marked at 37°C (Fig 3D).

### GalE presence and activity in *E*. *histolytica* trophozoites

**Fig 5:**
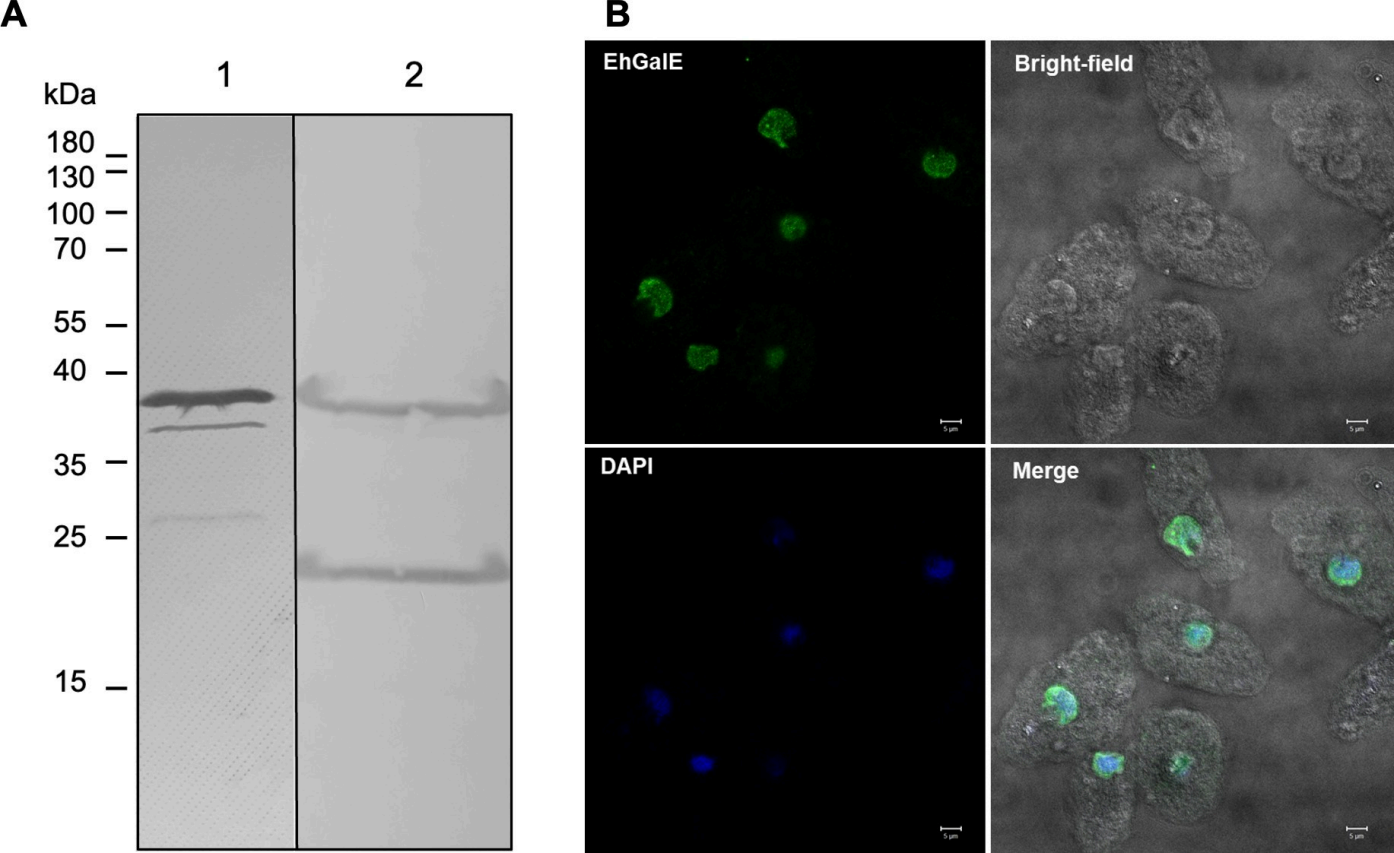
Presence and localisation of GalE protein in *E*. *histolytica* trophozoites. (A) GalE protein expression was visualised by western blot analysis with an *E*. *histolytica* specific anti-GalE antibody. *Lane 1*: recombinant *E*. *histolytica* GalE *Lane 2*: GalE in extract of *E*. *histolytica* trophozoites. (B) For immunofluorescence microscopy, *E*. *histolytica* trophozoites were fixed with 4% paraformaldehyde, permeabilized with 0.1% saponin and incubated with anti-GalE rabbit antiserum (1:100) overnight. For visualisation, a secondary anti-rabbit antibody conjugated to Alexa 488 (1:500) was added to the cells for 1 hour. Finally, cells were stained with 1 μg/ml DAPI. Pictures were obtained with an inverted Zeiss (LSM 700) confocal microscope, scale bar: 5 μm. The figure shows a representative of three experiments.

The native EhGalE was also examined biochemically. Firstly, a *E*. *histolytica* specific, affinity-purified anti-peptide antiserum against EhGalE was used to visualise the presence of the GalE protein in the trophozoites (Fig 5A). As described above, the protein is predicted to have a molecular mass of 37.8 kDa (Lane 2), in comparison to 39.3 kDa of the recombinant EhGalE, a discrepancy due to the additional Strep-tag and linker (Lane 1). We noted that GalE in the extract from trophozoites displayed a distinct second band with an estimated molecular mass of about 15 kDa less than the full-length protein. As the epitope of the anti-GalE antiserum is close to the amino-terminus, the smaller fragment is not visible on the blot. Also the recombinant GalE preparation showed smaller bands. So far we do not know why both recombinant and natural GalE displayed these varying degradation phenomena.

Our affinity-purified anti-GalE antiserum was then used to localise EhGalE. Trophozoites were grown under anaerobic conditions, fixed and incubated with a 1:100 dilution of the antiserum. Bound antibodies were visualised with a 1:500 dilution of Alexa 488 labelled secondary antibodies. Nuclei were stained with DAPI. The results are shown in Fig 5B. EhGalE was found in the cells, but quite unexpectedly, the stain was strongest around the nuclei. Although a cytoplasmic localisation may be expected from its metabolic function, a recent study on UGE1 (UDP-glucose 4-epimerase-1) in rice root hair cells reported its presence in nuclei as well as in the endoplasmic reticulum and cell membranes [40]. When we incubated *E*. *histolytica* trophozoites only with the secondary antibody, we did not observe any staining, only the DAPI stain was seen (S5 Fig).

To demonstrate not only the presence but also the activity of GalE, *E*. *histolytica* trophozoites were harvested and the lysate was examined with the same coupled enzyme assay as described above. Specific activity of GalE in the trophozoites was determined to be 3.12 ± 1.36 nmol/min/mg.

### EhGalE can epimerise multiple substrates

**Fig 6:**
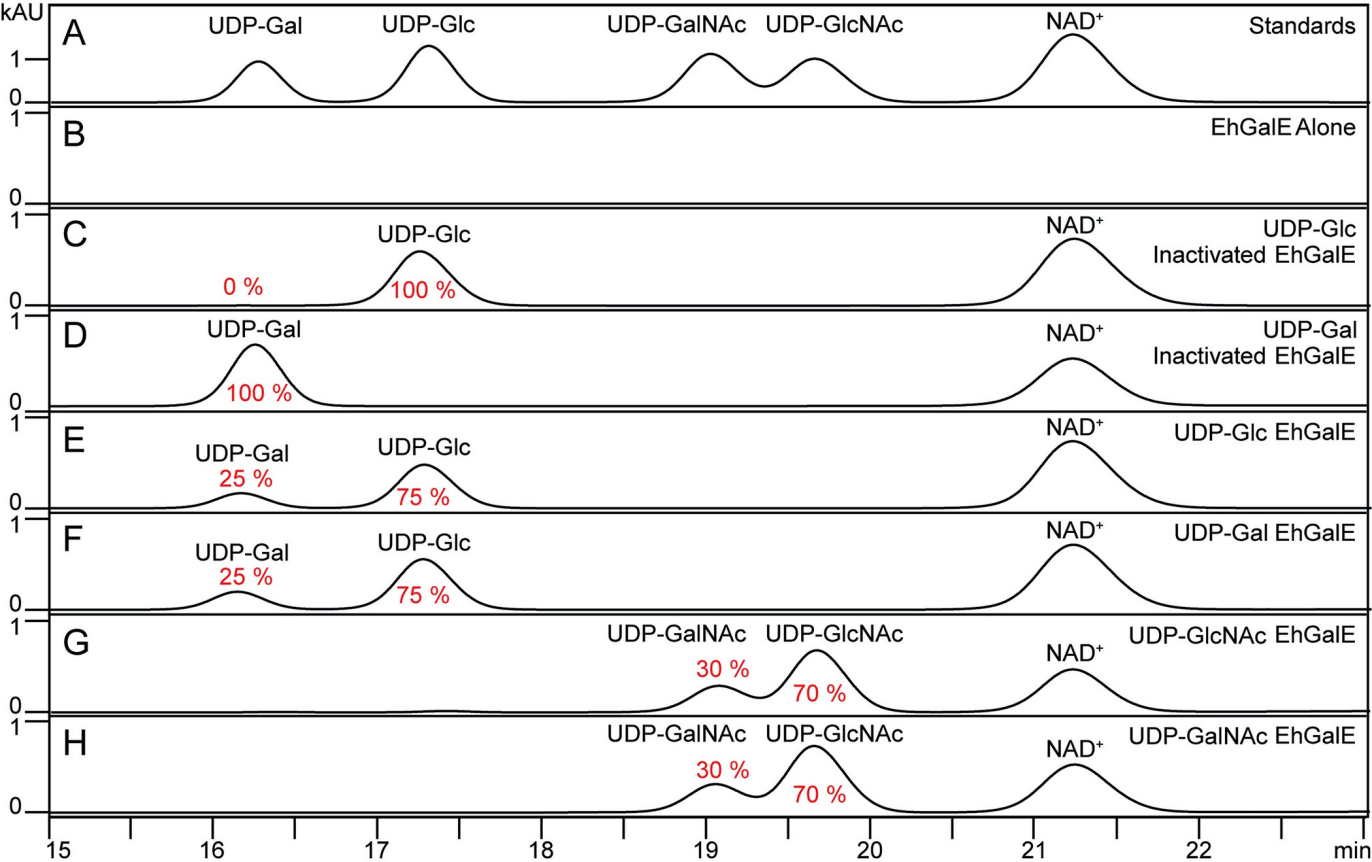
RP-HPLC chromatograms demonstrate various substrates of EhGalE. (A) Nucleotide sugars as standards and NAD^+^. (B) EhGalE without NAD^+^ and nucleotide sugar. (C-D) Inactivated (5 min at 95°C) EhGalE with NAD^+^ and UDP-glucose or UDP-galactose. (E-H) EhGalE was incubated for 1 h at 37°C with indicated nucleotide sugars and NAD^+^. Chromatograms were acquired by measuring at 254 nm, only minutes 15–24 of the chromatograms are displayed. Percentage of sugar conversion is indicated below or above the corresponding peak.

After the kinetic characterisation of EhGalE using the coupled assay, reversed-phase high-performance liquid chromatography (RP-HPLC) was used to elucidate its substrate specificity. As depicted on Fig 6E and 6F, EhGalE was confirmed to be able to epimerise UDP-Gal to UDP-Glc reversibly. After 1 hour of enzymatic reaction, the reaction equilibrium of 25% UDP-Gal and 75% UDP-Glc was reached, a ratio similar to that found in studies on other GalE enzymes [41]. Furthermore, it was tested whether EhGalE can also epimerise UDP-GalNAc to UDP-GlcNAc and vice versa (Fig 6G and 6H). As no UDP-GlcNAc dehydrogenase for a coupled assay was available, these substrates could only be investigated by RP-HPLC. Here the equilibrium slightly differed to the previous reactions with UDP-Gal and UDP-Glc; after 1 hour the reaction contained 30% UDP-GalNAc and 70% UDP-GlcNAc. Thus, EhGalE is able to use UDP-Glc, UDP-Gal, UDP-GlcNAc and UDP-GalNAc as substrates.

### EhGalE is inhibited by ebselen but not by diethylstilbestrol or ethacrynic acid

**Fig 7:**
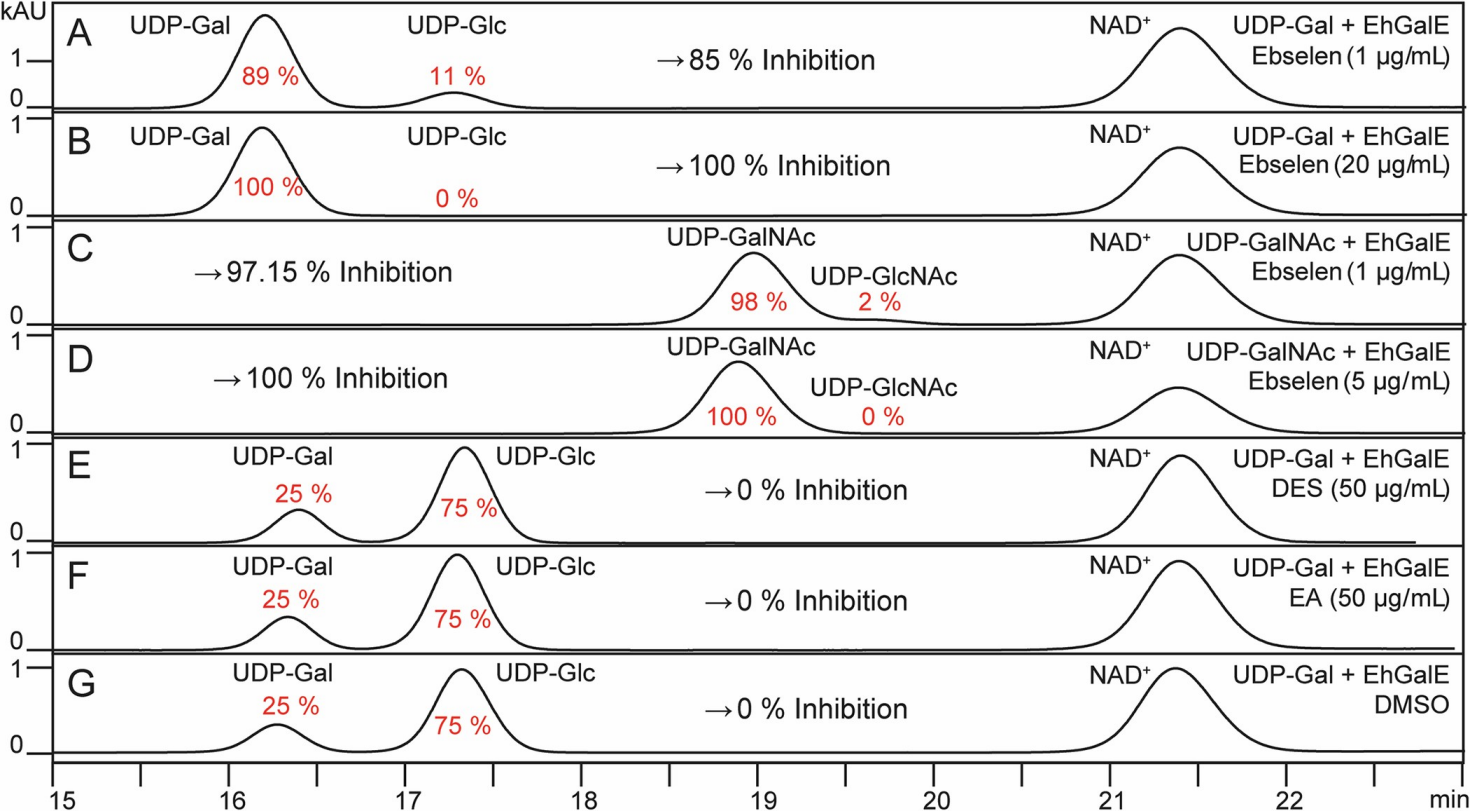
RP-HPLC chromatograms illustrate inhibition of EhGalE by ebselen but not DES or EA. (A) and (B) EhGalE was treated 30 min with 1 or 20 μg/ml ebselen, respectively, before addition of UDP-Gal. (C) and (D) EhGalE was treated 30 min with 1 or 5 μg/ml ebselen, respectively, before addition of UDP-GalNAc. (E-G) EhGalE was treated 30 min with 50 μg/ml diethylstilbestrol (DES) or ethacrynic acid (EA) or dimethyl sulfoxide as negative control (DMSO), respectively, before addition of UDP-Gal. Chromatograms were acquired by measuring at 254 nm, only minutes 15–24 are displayed. Percentage of sugar conversion is indicated below or above the corresponding peak, and percentage of inhibition is shown before or after the sugar peaks. The ebselen inhibition experiment for the conversion of UDP-Glc and UDP-Gal was repeated twice.

For determination of a potential effect of the compounds ebselen, diethylstilbestrol (DES) or ethacrynic acid (EA) on EhGalE, RP-HPLC was again the method of choice. The photometric method could not be used as the inhibitors were also active against the coupled enzyme UGDH. The conversion of UDP-Gal to UDP-Glc by EhGalE was inhibited by 84.93% with 1 μg/ml ebselen and by 100% with 20 μg/ml ebselen (Fig 7A and 7B, respectively). The IC50 of ebselen against the EhGalE substrate UDP-Gal was determined from three series of HPLC runs using the ebselen concentrations 0.2, 0.5, 1, 5 and 20 μg/ml, resulting in an IC50 of 2.19 μM with a geometric standard deviation of 1.15 (range 1.89–2.46 μM).

Similar inhibition was observed when using the substrate UDP-GalNAc (Fig 7C and 7D), where 1 μg/ml ebselen led to 97.15% and 5 μg/ml to 100% inhibition of EhGalE. Here, the IC50 was determined with the ebselen concentrations 0.2, 0.5, 1 and 5 μg/ml to be 1.2 μM. For technical reasons, only one set of HPLC runs was performed.

In contrast to ebselen, DES and EA were not able to inhibit the activity of EhGalE, not even at a concentration as high as 50 μg/ml (Fig 7E and 7F). Also, DMSO, in which ebselen, DES and EA are dissolved, does not have any influence on EhGalE activity (Fig 7G). Thus, these results suggest that of the three compounds tested, ebselen is the only, but very potent, EhGalE inhibitor.

### Ebselen does not inhibit *E*. *histolytica* cysteine proteases

**Fig 8:**
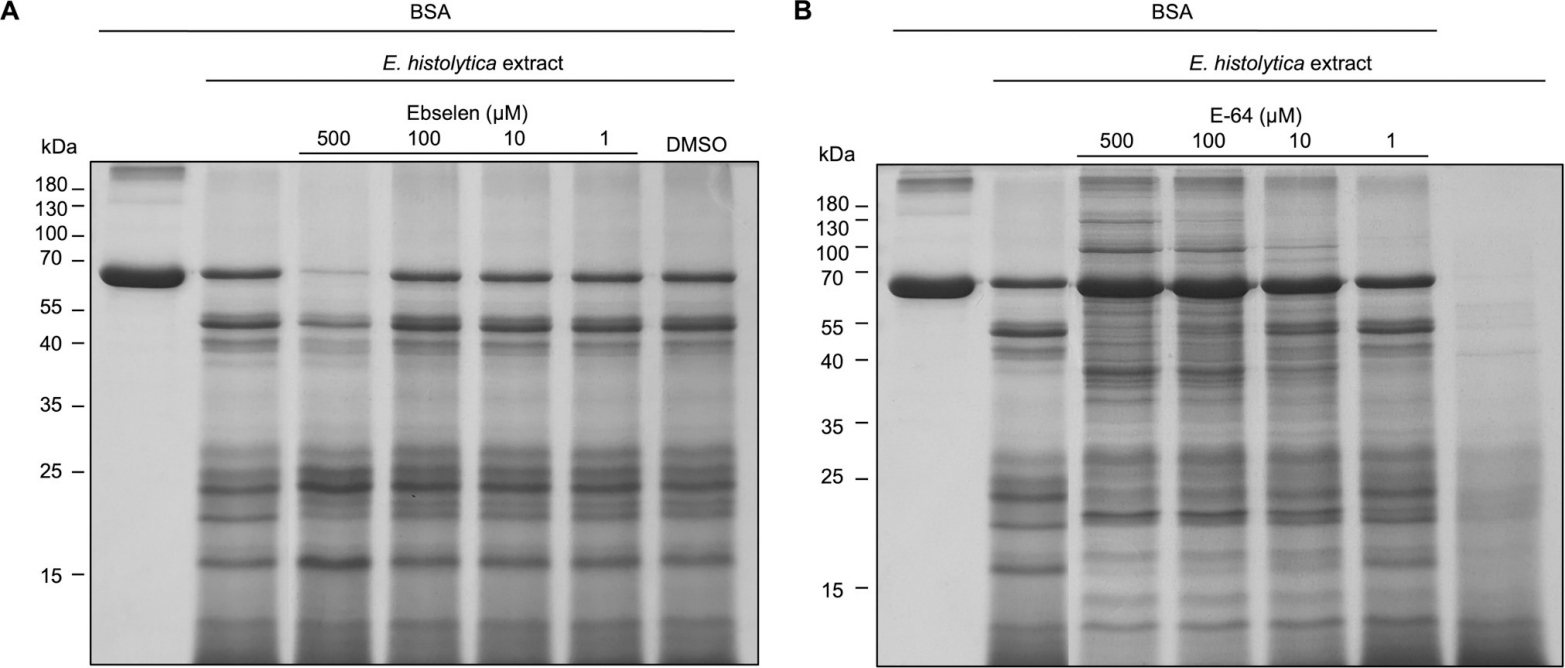
Ebselen does not inhibit protease activity in *E*. *histolytica* extracts. Trophozoite extract of *E*. *histolytica* was added to 10 μg of BSA. (A) Various concentrations (1–500 μM) of ebselen did not inhibit the degradation of BSA by proteases in the cell extract, the highest concentration even appeared to stimulate protease activity. The ebselen solvent DMSO has no effect on the reaction. (B) As a control, E-64, a cysteine protease inhibitor, blocks the cleavage of BSA starting at a concentration of 10 μM E-64.

Ebselen is a compound with various activities and one of them is the inhibition of cysteine proteases such as the SARS-CoV-2 main protease [42]. *E*. *histolytica* is a parasite rich in protease activity and cysteine proteases play a major role [43]. Therefore, it was interesting to test if ebselen inhibited *E*. *histolytica* cysteine proteinases. Due to the lack of purified or recombinant cysteine proteases from this species, only an experiment with *E*. *histolytica* trophozoite extract was performed using the substrate bovine serum albumin (BSA) in the presence or absence of ebselen (Fig 8A). The *E*. *histolytica* extract containing proteases cleaved BSA (66 kDa) into various fragments with molecular masses of around 50 kDa and between 15 and 25 kDa. Ebselen in concentrations up to 100 μM clearly did not inhibit the degradation of BSA by the cell extract. At the highest concentration of 500 μM, it appeared as if, unexpectedly, ebselen stimulated rather than inhibited *E*. *histolytica* protease activity. For comparison, the experiment was carried out with the cysteine protease inhibitor E-64 (Fig 8B), which diminished the degradation BSA in a concentration-dependent manner. However, even at a concentration of 500 μM E-64, some degradation of BSA was observed, probably due to other proteases such as serine proteases or metalloproteases. Taken together, unlike E-64, ebselen did not inhibit *E*. *histolytica* proteases, and it appears that at the highest concentration ebselen even stimulated rather than inhibited protease activity.

### Activity of ebselen, diethylstilbestrol and ethacrynic acid against *E*. *histolytica* trophozoites

**Fig 9:**
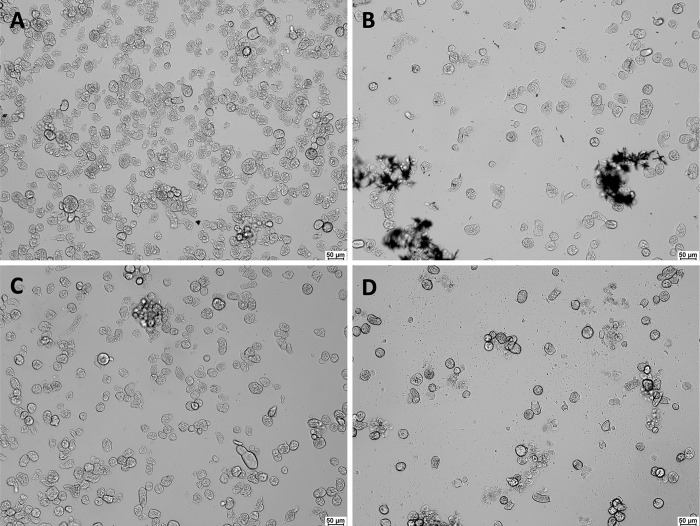
Effect of 24 h incubation with various inhibitors against *E*. *histolytica* culture. (A) Control trophozoites. (B) Trophozoites treated with 25 μg/ml (91.2 μM) ebselen. (C) Trophozoites treated with 25 μg/ml (93.2 μM) DES. (D) Trophozoites treated with 25 μg/ml (82.5 μM) EA. Photographs of the parasites were taken on a Leica DM IL LED inverted light microscope at 100x magnification. Scale bar = 50 μm.

**Fig 10:**
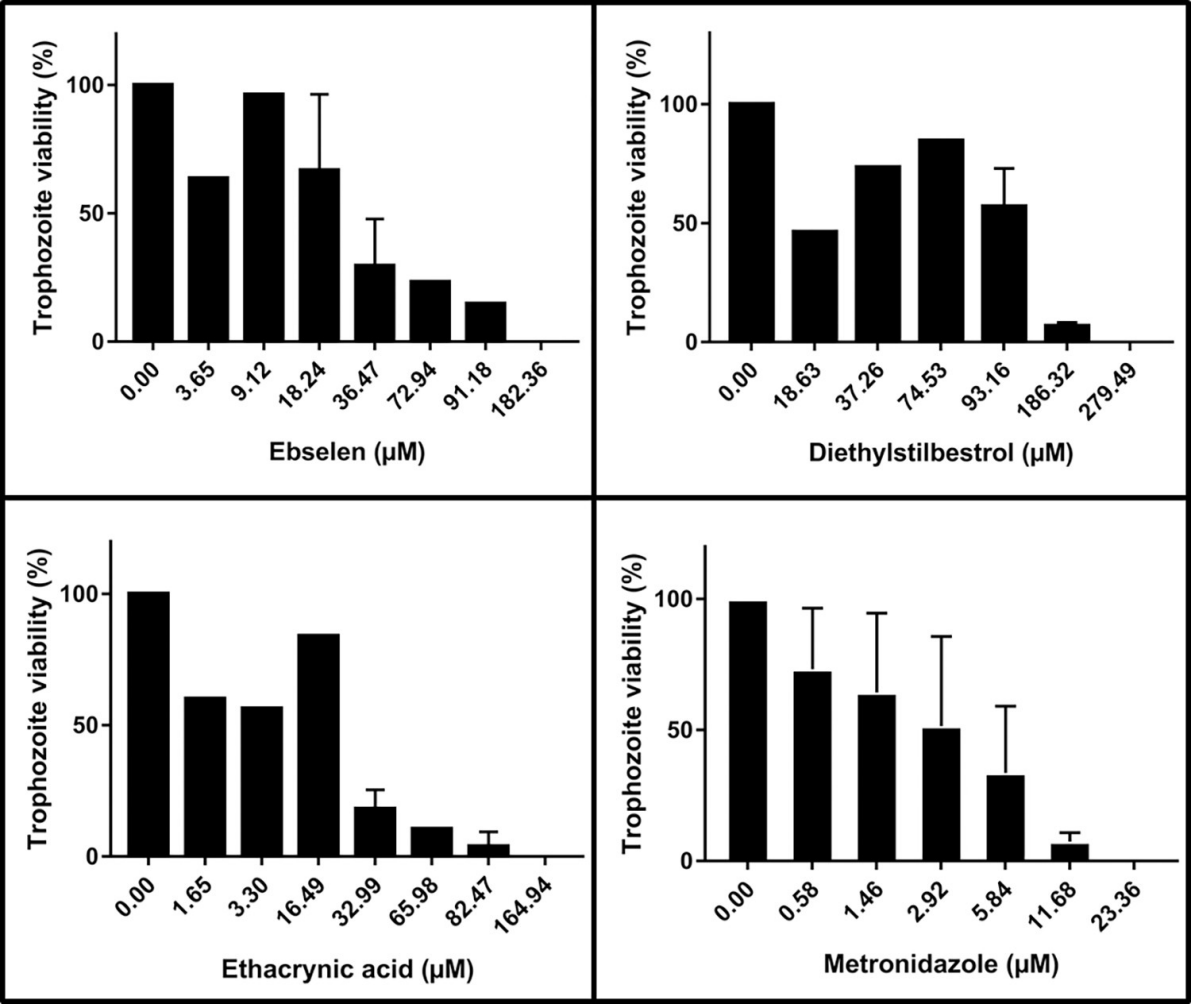
Viability of *E*. *histolytica* trophozoites. Trophozoites were incubated in 96-well plates for 24 h with an increasing concentration of ebselen, diethylstilbestrol, ethacrynic acid or metronidazole. Living cells were stained with aqueous eosin (0.5%) for quantification. All experiments were performed at least twice in duplicate.

To examine the effect of the three putative inhibitors on *E*. *histolytica* culture, the trophozoites were incubated for 24 h with an increasing concentration of each compound and changes of the phenotype or activity were observed under the microscope. As seen on Fig 9, at a concentration of 25 μg/ml all three compounds have a visible effect on trophozoites growth and viability. The largest effect on the cell viability was observed with EA, followed by ebselen (Fig 10). Thus, ebselen not only has an inhibitory effect on EhGalE but also on the *E*. *histolytica* culture. As ebselen precipitates at >25 μg/ml, its effect may be more limited at this concentration than EA (Fig 9B). DES and EA have a suppressing effect against culture growth and viability as well, however, appear to have a different target than EhGalE.

The effects of the three compounds were semi-quantitatively assessed by treating the trophozoites with varying concentrations of the inhibitors under anaerobic conditions and staining the living trophozoites with aqueous eosin (0.5%) (Table 1). Table 1 shows the EC50 values [μM] with geometric standard deviations σ_G_ as well as the minimum amoebicidal concentrations (MAC) (at which 99.99% of the culture is dead). Expectedly, the standard drug metronidazole had the highest effect, a modest effect was observed for ebselen and EA, while the effect of DES was weaker.

**Table 1:** EC50 with geometric standard deviations σ_G_ and ranges as well as minimum amoebicidal concentration (MAC) of three *E*. *histolytica* inhibitors. The values were determined by incubation of the trophozoites with increasing concentrations of the respective inhibitor for 24 h. Two biological repeats with two repeated series of concentrations were carried out.

Compound	EC50 [μM]	Geometric standard deviation [σ_g_]	Range [μM]	MAC [μM]
Ebselen	49	1.4	34–70	182
Diethylstilbestrol (DES)	110	1.8	59–204	279
Ethacrynic acid (EA)	14	2.0	7–27	165
Metronidazole	3.1	2.5	1.3–7.7	23

## Discussion

In this study, *E*. *histolytica* GalE was expressed in *E*. *coli* and purified by Strep-tag affinity chromatography. To our knowledge, this technology had been used only once before in *E*. *histolytica* studies to purify a protein expressed in this parasite [44]. The parameters K_m_, V_max_, as well as temperature and pH optima were measured for the interconversion of UDP-Gal and UDP-Glc, using an enzyme assay coupling the GalE reaction to UDP-glucose dehydrogenase. The temperature optimum of EhGalE was 37°C as the human GalE, however the pH optimum of EhGalE was 8.5 in comparison to 9.5 of the human GalE [45]. The K_m_ of EhGalE for the substrate UDP-Gal was determined to be 32 μM, which is not dissimilar to those defined for the human GalE between 48 μM [46] and 69 μM [47], and the *Trypanosoma brucei* GalE with a K_m_ of between 77 μM [20] and 95 μM [21].

We discovered that EhGalE cannot only interconvert UDP-Glc and UDP-Gal but can also act on UDP-GlcNAc and UDP-GalNAc as substrates. *E*. *histolytica* possesses a heterodimeric β-*N*-acetylhexosaminidase [13], which can cleave both GlcNAc and GalNAc from the colonic Muc2. In the next steps, these molecules could be taken up and phosphorylated in the 1-position. Then they could be reacted with UTP to form UDP-GlcNAc and UDP-GalNAc. Only UDP-GlcNAc can serve as a precursor of chitin when the trophozoites differentiate into cysts. The ability to epimerise UDP-GalNAc to UDP-GlcNAc allows *E*. *histolytica* GalE to produce more building blocks for chitin. The UDP-GlcNAc can then be transported into the Golgi apparatus for chitin synthesis by a UDP-GlcNAc transporter which has been characterised in *E*. *histolytica* [48]. Interestingly, contact of *E*. *histolytica* with human colon explants was not only associated with increased expression of mRNAs encoding the Gal/GalNAc lectin and cysteine proteases, respectively involved in binding and degradation of human mucins, but also of GalE and other glycan-metabolising enzymes [49]. Taken together, it is reasonable to hypothesise that EhGalE may not only play a role in the catabolism of galactose but also in the anabolism of the cyst chitin. Additionally, galactose is a key residue of the N-glycans [15] and LPPG [9] of *E*. *histolytica*, but technical challenges meant that we could not construct a knock-down strain in order to directly prove the necessity of GalE for the biosynthesis of these glycoconjugates.

**Fig 11:**
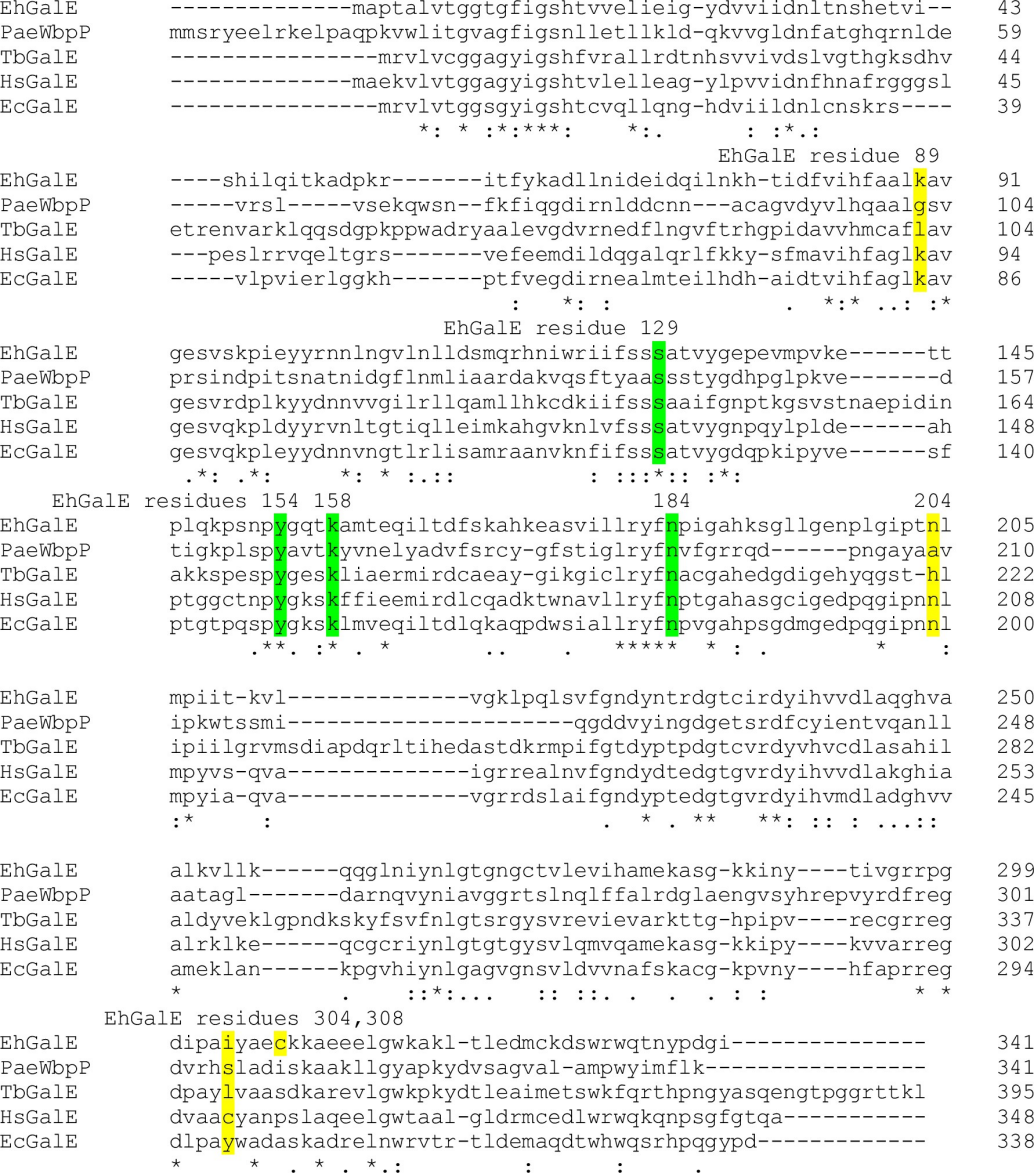
Alignment of EhGalE with important GalE homologs. EhGalE (accession XP_650346) was aligned with the GalE homologs WbpP (accession AAM27817) from *Pseudomonas aeruginosa* (Group 3) [26], TbGalE (accession XP_828356) from *Trypanosoma brucei* (Group 1a), HsGalE (accession Q14376) from *Homo sapiens* (Group 2), and EcGalE (accession AAC73846) from *E*. *coli* (Group 1b) using Clustal Omega (https://www.ebi.ac.uk/Tools/msa/clustalo/). Fully conserved residues important for activity [26] were marked in green. The substrate specificity determining residues were marked in yellow. Residues Gly102, Ala209 and Ser306 of WbpP determine its substrate specificity as group 3. The corresponding residues in EhGalE are Lys89, Asn204 and Ile304/Cys308 which classifies EhGalE as a Group 2 enzyme which agrees with our results.

As mentioned above, Ishiyama and colleagues [26] defined the three groups of GalE enzymes based on their substrate preferences and suggested, that the residues lining the hexagonal UDP-sugar binding site determine these preferences, which could be used for prediction of substrate specificity. The comparison of the sequences of EhGalE, *P*. *aeruginosa* WbpP, and other important GalEs are shown in Fig 11. The conserved residues Ser142, Tyr166, Lys170 and Asn195 in *P*. *aeruginosa* WbpP [26], corresponding to Ser129, Tyr154, Lys158 and Asn184 in EhGalE were marked in green. The residues Gly102, Ala209 and Ser306 of WbpP, marked in yellow, influence the space in the active site and predicted a Group 3 activity (only UDP-NAc sugars) for WbpP [26]. The corresponding residues in EhGalE were Lys89, Asn204 and Ile304/Cys308 which would correctly predict a Group 2 activity both on UDP-Glc/Gal and UDP-GlcNAc/GalNAc as for the human enzyme. The residues around the position 304/306 play the major role in the interaction with the UDP-sugars and thus have the largest influence on the substrate specificity. Mutant human GalE lacking Cys 307 lacks the ability to work on UDP-GlcNAc/GalNAc, but can still epimerize UDP-Gal to UDP-Glc [50]. When the large residue Tyr299 of the *E*. *coli* enzyme is replaced by Cys, the enzyme acquires the ability to epimerise UDP-GlcNAc/GalNAc [51]. GalE from *T*. *brucei* [20] and *T*. *cruzi* [52] are Group 1 enzymes [26] working on UDP-Gal/Glc only, and they have bulky Leu residues in the respective position. The trypanosomatid parasites do not incorporate UDP-GalNAc into their abundant glycoconjugates and therefore they do not need this particular GalE activity [53]. The *Giardia intestinalis* Group 3 enzyme [37] like *P*. *aeruginosa* WbpP possesses a Cys297. Unlike *E*. *histolytica*, *G*. *intestinalis* incorporates GalNAc for the biosynthesis of its cyst material [54], and both *E*. *histolytica* and *G*. *intestinalis* may use their GalE to interconvert the *N*-acetylamino sugars cleaved from the host mucin for use in their cyst wall. Whereas most residues in the consensus sequences proposed by Ishiyama align exactly, this is not exactly the case for all the described residues determining the specificity. So the *E*. *histolytica* Cys308 is four residues downstream and *G*. *intestinalis* Cys297 is three residues upstream from the exact alignment. Therefore, a protein structure analysis incorporating the substrates is needed to fully understand the enzymatic activity of the GalE homologues.

The compounds ebselen, DES and EA were reported as inhibitors of *T*. *brucei* GalE and were active against the parasite [21]. Therefore, the compounds were tested against *E*. *histolytica* GalE and against the trophozoites. A technical problem was encountered for the spectrophotometer assay that the helper enzyme UGDH was inhibited by all three compounds. Therefore, the susceptibility of EhGalE to the compounds was tested using RP-HPLC to determine the products directly. Clearly, EhGalE was not inhibited by DES and EA. Only ebselen suppressed the activity of EhGalE with an IC50 of 1.79 μM. As a comparison, *T*. *brucei* GalE was inhibited by ebselen with an IC50 of 0.62 μM.

In contrast to the effect on EhGalE, all three inhibitors were cytotoxic against *E*. *histolytica* trophozoites. An ebselen concentration of 182 μM (50 μg/ml) killed all trophozoites, however, some precipitation of the drug was observed already at above 20 μg/μl (Fig 9B). In contrast to the eosin measurements with metronidazole, the measurements of the three other inhibitors yielded more scattered results (Fig 10). The EC50 value for ebselen was estimated with GraphPad Prism as 49 μM. So, the effect on *E*. *histolytica* is significantly weaker than against other parasites: bloodstream-form *T*. *brucei* were inhibited with an EC50 of 4.1 μM [21]. *Naegleria fowleri*, the “brain-eating” amoeba, was sensitive to ebselen with an EC50 of 6.1 μM after 48 h of incubation [55]. *Plasmodium falciparum* was sensitive to ebselen with an EC50 6.8 μM [56], *Leishmania major* was sensitive with an EC50 of 4.1 μM and *T*. *brucei* was confirmed to be sensitive [57]. Both studies, however, suggested hexokinases as targets of ebselen. Unrelated heterocyclic or aromatic compounds with nM/μM IC50 values were shown to inhibit the *Fasciola hepatica* GalE [58].

As early as 1953, it was reported that DES inhibited the growth of *Entamoeba invadens* but not *Acanthamoeba castellanii* [59]. Bloodstream-form *T*. *brucei* were inhibited by DES with an EC50 of 2.8 μM [21]. The effect against *E*. *histolytica* with an EC50 of 110 μM was the weakest of all inhibitors. EA inhibited bloodstream-form *T*. *brucei* with an EC50 of 3.3 μM [21]. In our studies EA did not inhibit EhGalE activity and displayed an estimated EC50 of around 14 μM and a MAC of 165 μM. Taken together, of the three putative GalE inhibitors, only ebselen inhibited the *E*. *histolytica* enzyme, however, all three inhibitors inhibited the growth of the *E*. *histolytica* trophozoites to a varying extent.

In conclusion, our data show that, in addition to its substrate preference, *E*. *histolytica* GalE is phylogenetically more similar to the human enzyme than those characterised from other unicellular parasites, suggesting that developing a specific inhibitor of the *E*. *histolytica* enzyme may be a challenge. Furthermore, one can hypothesise that in the colon environment, the human host and the trophozoites use the same *N*-acetylated sugars for their purposes, to either make mucins or a chitin cell wall, and that GalE plays an important role in both the host and parasite.

## Materials and methods

### Sequence analysis and phylogeny reconstruction

Protein homologues (194) of UDP-glucose 4-epimerase were downloaded from the UniProt database (November 2022) in FASTA format [29]. To find the most representative protein sequences we focused at first to sequences with confirmed biochemical function: Mammalian, *Homo sapiens* GalE (A0A384NL38); Insect, *Drosophila melanogaster* GalE (Q9W0P5); Trematoda, *Schistosoma japonicum* (Q5D9E1); Eurotiomycetidae, *Aspergillus fumigatus* UGE5 (Q4WV46), UGE4 (Q4WQU9), UGE3 (Q4WX18); Brassicales, *Arabidopsis thaliana* UGE1 (Q42605), UGE2 (Q9T0A7), UGE3 (Q8LDN8), UGE4 (Q9C7W7), UGE5 (Q9SN58); Pseudomonas, *Pseudomonas aeruginosa* (Q9I3W0); Trichomonadida, *Trichomonas vaginalis* (A2FQB4); Kinetoplastea, *Trypanosoma cruzi* (Q4DN47); Diplomonadida, *Giardia intestinalis* (Q868I5); Amoebozoa, *E*. *histolytica* GalE (C4M746). The list of well-known protein sequences aided identification of more homologues from selected groups of organisms, based on the available annotation in the UniProt database. Data were retrieved after comparing a phylogeny tree of homologues and finally sequences were chosen which were used to generate the final phylogeny tree.

All alignments were performed with the MAFFT algorithm [31] after using trimAl [33]. In a final step IQTREE version 1.6.12 [30] was used to generate the Maximum-likelihood tree. The resulting phylogeny tree was visualized with iTOL [32] and monophyletic branches were collapsed to simplify the tree.

### Parasite culture

*E*. *histolytica* HM-1:IMSS-B was anaerobically cultured at 37°C in TYI-S-33 medium [60] supplemented with 10% (v/v) complement inactivated, bovine serum (Capricorn Scientific, Germany) and 1% (v/v) penicillin/streptomycin solution (10,000 units penicillin and 10 mg streptomycin/ml, Sigma-Aldrich, USA) and 3% (v/v) of vitamin mixture (Diamond Vitamin Tween 80 Solution, SAFC Biosciences, KA, USA). The *E*. *histolytica* trophozoites were sub-cultured in 12.5 cm^2^ tissue culture flasks (Corning, USA) twice a week under sterile conditions.

### Recombinant expression and purification of EhGalE

The genome-derived sequence of *E*. *histolytica* GalE (XP_650346) was reverse-translated into a DNA sequence with the codon usage adapted to high expression in *E*. *coli* and chemically synthesized (BioCat, Heidelberg, Germany). At the amino-terminus, the coding sequence for the Strep-tag Trp-Ser-His-Pro-Gln-Phe-Glu-Lys was added for the expression from the plasmid pET-17b (Merck, Darmstadt, Germany). Because the tag sequence starts with a Trp residue, which may negatively influence the expression [61], the residues Ser Ala were added after the initiator Met, and downstream of the tag, we added the residues Gly-Gly-Gly-Ser as a flexible linker. The whole construct is shown in S3 Fig. The construct for the expression of human UGDH for the coupled enzyme assay is displayed in S4 Fig.

EhGalE expression was obtained by transformation of plasmid pET-17b into BL-21AI *E*. *coli* (Invitrogen, Massachusetts, United States) and induction with 0.2% (w/v) L-arabinose. After 2.5 hours of growth, the bacteria were harvested by centrifugation at 4,500 x g for 12 min at 4°C, frozen in liquid nitrogen, and disrupted with a mortar. The crude lysate was cleared by centrifugation (14,000 rpm, 10 min, 4°C) and EhGalE was purified on a gravity flow Strep-Tactin Sepharose column (IBA Lifesciences, Göttingen, Germany).

The obtained fractions were analysed by a 12.5% SDS-polyacrylamide gel electrophoresis. To estimate the size of the protein, 5 μl of a protein ladder (Page Ruler, Thermo Scientific) were loaded on the gel. The 3 fractions with the highest amount of eluted enzyme were pooled and protein concentration was determined by the Bradford assay (Bio-Rad).

### Enzyme characterisation and kinetic parameters

The activity of recombinant EhGalE was measured in a Lambda 25 UV/VIS spectrophotometer (Perkin Elmer, USA) by monitoring the coupled assay (Fig 4) measuring the increase of NADH at λ = 340nm (Δɛ_340_ = 6.2 mM^−1^ cm^−1^) catalysed by the enzyme UDP-glucose dehydrogenase (UGDH). 1 μg of EhGalE was added to the standard reaction buffer containing 0.1 M HEPES pH 8.5, 2.5 mM NAD^+^, 0.4 mM UDP-galactose and 2 μg UGDH.

The pH dependence of EhGalE was obtained at 37°C using two buffers: 0.1 M MES for the measurements of pH 6–7 and 0.1 M HEPES for the pH range 7.5–9.

The optimum temperature of EhGalE was determined in the standard reaction buffer at the temperatures 15, 20, 25, 37 and 42°C. To assure the right temperature, the reaction buffer was incubated in the pre-heated spectrophotometer for 5 minutes before the addition of EhGalE and the start of the measurement.

Michaelis-Menten constants were determined by adding 1 μg of the recombinant EhGalE to 0.1 M HEPES pH 8.5 buffer, containing 2.5 mM NAD^+^, 2 μg of UDP-glucose dehydrogenase (UGDH) and the substrate UDP-galactose in increasing concentrations (0.05 mM, 0.1 mM, 0.2 mM and 0.4 mM).

All EhGalE activity measurements were performed in triplicates over a period of 5 minutes at 37°C and OD values were noted every 30 seconds.

### RP-HPLC

The activity of EhGalE was examined on a Reversed Phase HPLC (Shimadzu, Japan) using a COSMOSIL 5C18-AR-II packed column (Nacalai Tesque, Japan) at RT with absorbance set at 254 nm to detect nucleotide sugars. The HPLC gradient was based on buffer A (100 mM potassium phosphate pH 6.4 supplemented with 8 mM tetrabutylammonium hydrogen sulfate in HPLC grade H_2_O) and Buffer B (70% buffer A and 30% acetonitrile) as follows: 100% buffer A for 13 min; 0–77% linear gradient of buffer B for 22 min; 77–100% buffer B for 1 min; and 100% buffer B for 14 min using a constant flow rate of 0.8 ml/min [62]. Peaks were identified by comparison with the retention times of a standard mixture injected before the analysis. Each nucleotide sugar peak was integrated and quantified based on the peak areas of the calibration curve of each standard and was further identified using MALDI-ToF MS. Retention times were standardised against NAD^+^ peaks when a small shift was observed.

### Compounds

Ebselen and ethacrynic acid were purchased from abcr GmbH (Karlsruhe, Germany), dimethyl sulfoxide (DMSO), diethylstilbestrol and metronidazole from Sigma-Aldrich (USA). All inhibitors were dissolved in DMSO at 7.5 mg/ml and subsequently further diluted in DMSO.

### Inhibition of recombinant EhGalE

To examine the potential inhibition of EhGalE by the three compounds, EhGalE was incubated for 30 min with 20 μg/ml ebselen, diethylstilbestrol or ethacrynic acid together with 0.125 M HEPES pH 8.5 buffer containing 1.5 mM NAD^+^ and 1 μg EhGalE. Subsequently, the substrates UDP-Gal (4.5 mM) or UDP-GalNAc (1.5 mM) were added for an incubation of 1 hour at 37°C. Prior injection into the HPLC, the enzymatic reaction was inactivated for 10 min at 95°C and 1:10 diluted with the HPLC buffer A. Percentage of inhibition was calculated from the peak areas by comparison of treated and untreated samples, considering the reaction equilibrium. The determination of the IC50 of ebselen, was performed with the concentrations 0.2, 0.5, 1, 5 and 20 μg/ml.

### Cysteine protease inhibition assay

*E*. *histolytica* trophozoites were cultivated for 48 h and disrupted with a dounce homogenizer to prepare a cell extract, which had a protein concentration of 4.9 μg/μl as measured by Bradford assay. Ten μl of the extract were incubated at 37°C for 30 min with 10 μg of BSA (Fermentas). Ebselen or the cysteine protease inhibitor E-64 (Sigma), were individually added to the reactions at the concentrations of 1, 10, 100 and 500 μM, resulting in a total reaction volume of 30 μl. As DMSO had to be used to dissolve ebselen, the corresponding amount of DMSO alone was used as a control. All the samples as well as BSA alone and the cell extract alone were analysed by SDS-PAGE, and stained with Coomassie brilliant blue R-250 (AppliChem).

### Western blotting

*E*. *histolytica* trophozoites were harvested 48 hours after splitting and washed once with 1 x PBS. The cell suspension was then frozen with liquid nitrogen and ground with pestle and mortar. Subsequently, cells were centrifuged at 14,000 rpm for 10 min at 4°C and the protein concentration of the supernatant was measured with the Bradford assay. 30 μg total protein of the cell suspension was loaded and separated on a 12% SDS-polyacrylamide gel. Protein transfer from the gel to the PVDF membrane was achieved by using a wet blotting system (all materials from Bio-Rad). Following the transfer, the membrane was blocked in 3% BSA and subsequently incubated in the primary antibody solution (1:1000 dilution in 3% BSA) containing a rabbit anti-peptide antiserum against *E*. *histolytica* GalE. The antiserum had been raised against the predicted GalE epitope Thr-Lys-Ala-Asp-Pro-Lys-Arg-Ile-Thr-Phe-Tyr-Lys-Ala-Asp, and the antibodies were affinity-purified (Biomatik, Wilmington, USA). The secondary alkaline phosphate-coupled goat anti-rabbit antibody (Merck, A3687) was then applied in a 1:10,000 dilution and finally, the blot was stained with 5-bromo-4-chloro-3-indolyl phosphate (BCIP) and nitro blue tetrazolium (NBT) for 5 min.

### Confocal immunofluorescence

*E*. *histolytica* trophozoites (100,000 cells) were grown for 24 h in a μ-Slide 4 well chamber (ibidi, Germany) in 300 μl TYI-S-33 medium and incubated at 37°C under anaerobic conditions in an air-tight box containing Anaerocult A (Merck). Subsequently, the medium was removed from the trophozoites, and cells were fixed with 4% (w/v) paraformaldehyde (Sigma) for 5 min, washed with TBST, permeabilized for 5 min in 0.1% (w/v) saponin (Sigma) and blocked in 1% BSA for 10 min. Cells were stained overnight with the anti-EhGalE antiserum (1:100 in 1% BSA), then washed with PBS. The secondary antibody, goat anti-rabbit conjugated to Alexa 488 (Thermo Fisher Scientific) (1:500 in 1% BSA), was applied to the cells for 1 h. To stain the nuclei, cells were incubated for 5–10 min with 1 μg/ml 4′,6-diamidino-2-phenylindole (DAPI) (Sigma) in H_2_O. After washing steps, images were obtained with the inverted confocal Zeiss LSM 700 microscope.

### *E*. *histolytica* cell extract activity measurement

*E*. *histolytica* culture was harvested like described above and the protein concentration of the supernatant was measured with the Bradford assay. 16.8 μg total supernatant protein containing the active EhGalE was immediately used for the activity measurements with the same standard reaction buffer as described above. Three independent measurements were performed in triplicates over a period of 5 minutes at 37°C and OD values were noted every 30 seconds.

### Inhibition of *E*. *histolytica* trophozoites / antiamoebic activity

The inhibition assays were carried out in 96-well plates (TPP, Trasadingen, Switzerland) that were kept in an air-tight box with Anaerocult A (Merck, Darmstadt, Germany), providing the necessary anaerobicity.

Assays were executed by seeding the trophozoites at a concentration of 12,000 cells/well (in 300 μl volume) and adding 3 μl of each compound (ebselen, diethylstilbestrol or ethacrynic acid) at indicated concentrations. Twenty-four hours later, cells were photographed on a Leica DM IL LED inverted light microscope. Subsequently, the medium was carefully removed by pipetting, cells were washed with PBS and fixed with 100 μl methanol. After removal of the methanol, 10 min staining with aqueous eosin solution (0.5%, Sigma) was performed [63]. Then, another washing step was executed and finally, 100 μl of PBS were added for absorbance measurements. The optical density of each well was detected by a micro plate reader (Anthos Labtec HT2) at 492 nm.

The EC50 (half maximal effective concentration) values were determined using GraphPad Prism. The minimum amoebicidal concentration (MAC) was defined as the concentration of the drug that leads to 99,99% of viability loss. The negative control was treated with 3 μl of DMSO, the solvent of all inhibitors. Metronidazole was added to the inhibitor assays as a positive reference compound. All conditions were performed at least twice in duplicate.

### Data analysis

Visualizations of the data, calculation of the kinetic constants and their standard deviations were performed in GraphPad Prism software 7.0. IC50 (half maximal inhibitory concentration of enzyme activity) was calculated by linear regression in Microsoft Excel 2016, and EC50 (half maximal inhibitory concentration of parasite growth) with geometric standard deviations and ranges was determined using GraphPadPrism software as well.

## Supporting information

S1 Fig Full phylogeny tree. Full phylogeny tree corresponding to collapsed phylogeny (shown in Fig 1) of 194 homologous protein sequences of UDP-glucose 4-epimerase from UniProt database. Numbers below the branches indicate bootstrap support (maximum value 100). Maximum-likelihood tree was based on the alignment of 764 columns with 468 parsimony-informative sites by IQTREE [30]. The LG+I+G4 model was automatically fitted by algorithm, alignment was done with using MAFFT [31], tree has been visualized with iTOL [32] and rooted at midpoint. In boxes indicated monophyletic branches with names of clades and name genes. Genes names were annotated from database based on well-known genes marked with bold font.
(SVG)

S2 Fig Unrooted phylogeny tree. Unrooted phylogeny tree of 216 homologous protein sequences of UDP-glucose 4-epimerase from UniProt database. Maximum-likelihood tree was based on the alignment of 331 columns with 294 parsimony-informative sites by IQTREE [30]. The LG+I+G4 model was automatically fitted by algorithm, alignment was done with using MAFFT [31] and trimAI [33]. Monophyletic branches have been marked with different colors corresponding to species groups and annotated with names based on well-known genes from the database.
(SVG)

S3 Fig EhGalE construct. The protein sequence of *E*. *histolytica* GalE (XP_650346) was reverse-translated into a coding sequence with *E*. *coli* codon usage for high expression. In the encoded sequence, the Strep-Tag for purification was added at the amino-terminus followed by a four-residue flexible linker. After the initiator methionine, the residues Ser-Ala were inserted to enhance the stability of the construct. The finished sequence was then added between the *Nde*I and *Xho*I sites of pET-17b.
(DOCX)

S4 Fig HsUGDH construct. In order to express the coupled enzyme for the GalE assay, the protein sequence of human UDP-glucose 6-dehydrogenase (HsUGDH, O60701) was reverse-translated into a coding sequence with *E*. *coli* codon usage for high expression. In the encoded sequence, the Strep-Tag for purification was added at the amino-terminus followed by a four-residue flexible linker. After the initiator methionine, the residues Ser-Ala were inserted to enhance the stability of the construct. The finished sequence was then added between the *Nde*I and *Xho*I sites of pET-17b.
(DOCX)

S5 Fig Confocal immunofluorescence with secondary antibody only. The immunofluorescence experiment was carried out without the primary rabbit anti-GalE antiserum. Nuclei were stained with DAPI as before.
(TIF)
