# Supplementary material for: Molecular characterisation of Entamoeba histolytica UDP-glucose 4-epimerase, an enzyme able to provide building blocks for cyst wall formation
Source: PLoS Negl Trop Dis. 2023 Aug 24;17(8):e0011574. doi: 10.1371/journal.pntd.0011574 (PMC10482301; doi:10.1371/journal.pntd.0011574)
Supplement: S3 Fig — The protein sequence of E. histolytica GalE (XP_650346) was reverse-translated into a coding sequence with E. coli codon usage for high expression. In the encoded sequence, the Strep-Tag for purification was added at the amino-terminus followed by a four-residue flexible linker. After the initiator methionine, the residues Ser-Ala were inserted to enhance the stability of the construct. The finished sequence was then added between the NdeI and XhoI sites of pET-17b. (DOCX) [file pntd.0011574.s003.docx]

NdeI

**cat**

**atg**agcgcatggagccatccgcagtttgaaaaaggtggcggtagcgcaccgaccgcactg
 M  S  A  **W  S  H  P  Q  F  E  K**  G  G  G  S  A  P  T  A  L 20
gttaccggcggtaccggttttattggtagtcataccgttgttgaactgattgaaattggc

 V  T  G  G  T  G  F  I  G  S  H  T  V  V  E  L  I  E  I  G 40

tatgatgtggttattatcgataatctgaccaatagtcatgaaaccgtgattagccatatt
 Y  D  V  V  I  I  D  N  L  T  N  S  H  E  T  V  I  S  H  I 60
ctgcagattaccaaagccgatccgaaacgtattaccttttataaagcagatctgctgaat
 L  Q  I  T  K  A  D  P  K  R  I  T  F  Y  K  A  D  L  L  N 80 
attgacgaaattgatcagattctgaacaaacataccattgattttgtgattcacttcgcc

I  D  E  I  D  Q  I  L  N  K  H  T  I  D  F  V  I  H  F A 100 
gccctgaaagcagtgggcgaaagtgtgagcaaaccgattgaatattatcgtaataacctg
 A  L  K  A  V  G  E  S  V  S  K  P  I  E  Y  Y  R  N  N L 120 
aatggcgttctgaatctgctggatagtatgcagcgtcataatatttggcgcattattttt
 N  G  V  L  N  L  L  D  S  M  Q  R  H  N  I  W  R  I  I  F 140
agcagcagtgccaccgtgtatggtgaaccggaagttatgccggttaaagaaaccaccccg
 S  S  S  A  T  V  Y  G  E  P  E  V  M  P  V  K  E  T  T  P 160
ctgcagaaaccgagcaatccgtatggtcagaccaaagcaatgaccgaacagattctgacc

L  Q  K  P  S  N  P  Y  G  Q  T  K  A  M  T  E  Q  I  L  T 180 
gattttagcaaagcacataaagaagccagtgttattctgctgcgttattttaatccgatt

D  F  S  K  A  H  K  E  A  S  V  I  L  L  R  Y  F  N  P I 200 
ggtgcccataaaagcggtctgctgggtgaaaatccgctgggcattccgaccaatctgatg

G  A  H  K  S  G  L  L  G  E  N  P  L  G  I  P  T  N  L  M 220
ccgattattaccaaagttctggtgggtaaactgccgcagctgagtgtgtttggtaatgat

P  I  I  T  K  V  L  V  G  K  L  P  Q  L  S  V  F  G  N  D  240
tataatacccgtgatggtacctgcattcgcgattatattcatgtggtggatctggcacag
 Y  N  T  R  D  G  T  C  I  R  D  Y  I  H  V  V  D  L  A  Q 260
ggtcatgtggccgcactgaaagttctgctgaaacagcagggtctgaatatctataatctg

G  H  V  A  A  L  K  V  L  L  K  Q  Q  G  L  N  I  Y  N  L 280 
ggcaccggtaatggctgcaccgtgctggaagttattcatgccatggaaaaagcaagcggt

G  T  G  N  G  C  T  V  L  E  V  I  H  A  M  E  K  A  S  G 300
aaaaagattaattacaccattgttggtcgccgcccgggcgatattccggcaatctatgcc

K  K  I  N  Y  T  I  V  G  R  R  P  G  D  I  P  A  I  Y A 320 
gaatgcaaaaaagcagaagaagaactgggttggaaagccaaactgaccctggaagatatg
 E  C  K  K  A  E  E  E  L  G  W  K  A  K  L  T  L  E  D  M 340

XhoI
tgcaaagatagctggcgttggcagaccaattatccggatggcatttaa**ctcgag**
 C  K  D  S  W  R  W  Q  T  N  Y  P  D  G  I  *   355
