# Supplementary material for: Molecular characterisation of Entamoeba histolytica UDP-glucose 4-epimerase, an enzyme able to provide building blocks for cyst wall formation
Source: PLoS Negl Trop Dis. 2023 Aug 24;17(8):e0011574. doi: 10.1371/journal.pntd.0011574 (PMC10482301; doi:10.1371/journal.pntd.0011574)
Supplement: S4 Fig — In order to express the coupled enzyme for the GalE assay, the protein sequence of human UDP-glucose 6-dehydrogenase (HsUGDH, O60701) was reverse-translated into a coding sequence with E. coli codon usage for high expression. In the encoded sequence, the Strep-Tag for purification was added at the amino-terminus followed by a four-residue flexible linker. After the initiator methionine, the residues Ser-Ala were inserted to enhance the stability of the construct. The finished sequence was then added between the NdeI and XhoI sites of pET-17b. (DOCX) [file pntd.0011574.s004.docx]

**NdeI**

**cat**

**atg**agtgcc**tggagtcatccgcagtttgaaaaa***ggtggcggtagc*tttgaaattaagaaa

M  S  A  **W  S  H  P  Q  F  E  K**  *G  G  G  S* F E I K K 20

atttgctgcatcggcgccggctatgttggtggtccgacctgcagtgtgattgcacacatg

I C C I G A G Y V G G P T C S V I A H M 40

tgtccggaaattcgcgttaccgttgtggatgtgaatgaaagccgcattaatgcatggaat

C P E I R V T V V D V N E S R I N A W N 60

agtccgaccctgccgatctatgaaccgggtctgaaagaagttgtggaaagttgtcgtggt

S P T L P I Y E P G L K E V V E S C R G 80

aaaaatctgtttttcagcaccaatattgacgatgccattaaggaagcagatctggttttt

K N L F F S T N I D D A I K E A D L V F 100

attagcgtgaataccccgaccaaaacctatggtatgggcaaaggccgcgccgcagatctg

I S V N T P T K T Y G M G K G R A A D L 120

aaatatattgaagcctgtgcacgtcgtattgtgcagaatagcaatggctataaaattgtg

K Y I E A C A R R I V Q N S N G Y K I V 140

accgaaaaaagcaccgttccggtgcgcgccgcagaaagcattcgccgcatttttgatgcc

T E K S T V P V R A A E S I R R I F D A 160

aataccaaaccgaatctgaatctgcaggtgctgagtaatccggaatttctggcagaaggt

N T K P N L N L Q V L S N P E F L A E G 180

accgccattaaggatctgaaaaatccggatcgcgttctgattggtggtgacgaaaccccg

T A I K D L K N P D R V L I G G D E T P 200

gaaggccagcgcgccgtgcaggcattatgcgcagtttatgaacattgggttccgcgcgaa

E G Q R A V Q A L C A V Y E H W V P R E 220

aaaattctgaccaccaatacctggagtagtgaactgagcaaactggccgcaaatgcattt

K I L T T N T W S S E L S K L A A N A F 240

ctggcccagcgcattagcagcattaatagtattagcgcactgtgtgaagcaaccggtgca

L A Q R I S S I N S I S A L C E A T G A 260

gatgttgaagaagttgcaaccgcaattggtatggatcagcgcattggtaataagtttctg

D V E E V A T A I G M D Q R I G N K F L 280

aaagcaagcgtgggctttggtggtagctgctttcagaaagatgtgctgaatctggtttat

K A S V G F G G S C F Q K D V L N L V Y 300

ctgtgtgaagccctgaatctgccggaagttgcacgctattggcagcaggttattgatatg

L C E A L N L P E V A R Y W Q Q V I D M 320

aatgattatcagcgccgccgctttgcaagccgtattattgatagcctgtttaataccgtg

N D Y Q R R R F A S R I I D S L F N T V 340

accgataaaaagattgcaattctgggctttgcattcaaaaaagataccggtgacacccgt

T D K K I A I L G F A F K K D T G D T R 360

gaaagtagcagcatctatattagtaaatacctgatggatgagggcgcccatctgcatatc

E S S S I Y I S K Y L M D E G A H L H I 380

tatgatccgaaagtgccgcgcgaacagattgttgttgatctgagccatccgggtgttagt

Y D P K V P R E Q I V V D L S H P G V S 400

gaagatgatcaggtgagccgcctggtgaccattagtaaagatccgtatgaagcatgtgat

E D D Q V S R L V T I S K D P Y E A C D 420

ggtgcccatgcagttgtgatttgtaccgaatgggatatgtttaaagaactggattatgaa

G A H A V V I C T E W D M F K E L D Y E 440

cgcattcataaaaagatgctgaaaccggcattcatttttgatggtcgccgcgtgctggat

R I H K K M L K P A F I F D G R R V L D 460

ggcctgcataatgaactgcagaccattggttttcagattgaaaccattggcaaaaaagtt

G L H N E L Q T I G F Q I E T I G K K V 480

agtagcaaacgtattccgtatgccccgagtggtgaaattccgaaattttcactgcaggat

S S K R I P Y A P S G E I P K F S L Q D 500

**XhoI**

ccgccgaataagaaaccgaaagtttaa**ctcgag**

P P N K K P K V * 508
