# Supplementary figures and images for: Molecular characterisation of Entamoeba histolytica UDP-glucose 4-epimerase, an enzyme able to provide building blocks for cyst wall formation
Source: PLoS Negl Trop Dis. 2023 Aug 24;17(8):e0011574. doi: 10.1371/journal.pntd.0011574 (PMC10482301; doi:10.1371/journal.pntd.0011574)

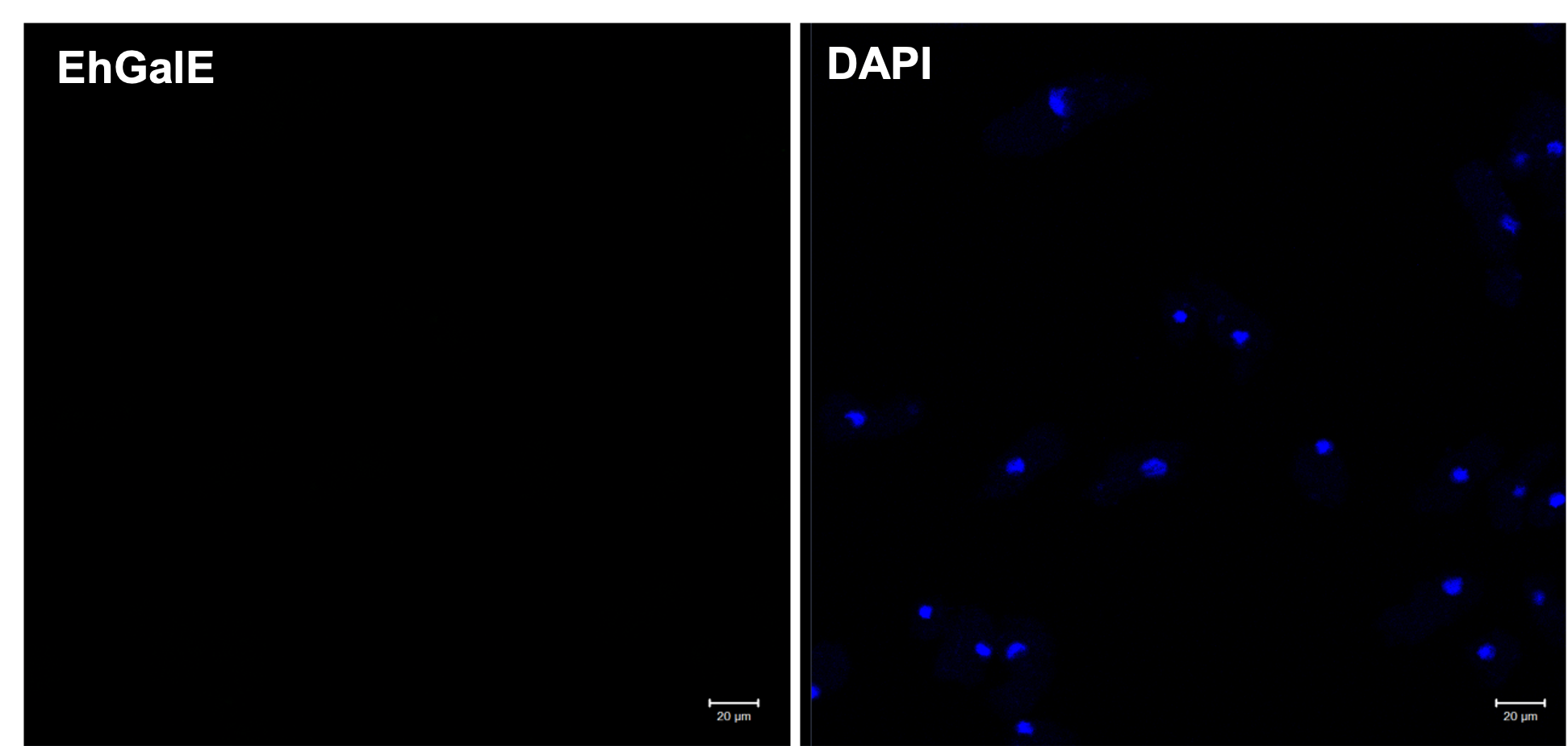

Supplement: S5 Fig — The immunofluorescence experiment was carried out without the primary rabbit anti-GalE antiserum. Nuclei were stained with DAPI as before. (TIF) [file pntd.0011574.s005.tif]
